# Activation of the intrinsic apoptosis pathway contributes to the induction of apoptosis in hepatocellular carcinoma cells by valproic acid

**DOI:** 10.3892/ol.2014.2739

**Published:** 2014-11-25

**Authors:** WEIHUA YANG, XIA ZHAO, FENGYAN PEI, MINGYU JI, WANSHAN MA, YUNSHAN WANG, GUOSHENG JIANG

**Affiliations:** 1 Central Laboratory, Jinan Central Hospital, Shandong University, Jinan, Shandong 250013, P.R. China; 2 Central Laboratory, Shandong Provincial Qianfoshan Hospital, Jinan, Shandong 250014, P.R. China; 3 Key Laboratory for Rare and Uncommon Diseases of Shandong Province, Key Medical Laboratory for Tumor Immunology and Chinese Medicine Immunology of Shandong Province, Institute of Basic Medicine, Shandong Academy of Medical Sciences, Jinan, Shandong 250062, P.R. China

**Keywords:** histone deacetylase inhibitor, hepatocellular carcinoma, valproic acid, apoptosis, cell cycle

## Abstract

The level of histone deacetylation is closely associated with the genesis and development of tumors, but the antitumor effect and mechanism of the class I histone deacetylase inhibitor (HDACI) valproate acid sodium (VPA) on hepatocellular carcinoma cells has not been clearly demonstrated. In the present study, the antitumor effect and mechanism of VPA were measured *in vitro*. Firstly, it was found that, as an HDAC inhibitor, VPA could inhibit HDAC activity and HDAC1 gene expression in hepatocellular carcinoma cells and, as a result, an inhibition of cell proliferation was detected by MTT assay. Subsequently, the cell cycle and cell apoptosis profiles were analyzed using flow cytometry (FCM). The expression of the mRNA and protein of cyclins A, D1 and E and P21^Waf/cip1^ was measured by reverse transcription-polymerase chain reaction and FCM analysis to determine the molecular mechanism of VPA-induced cell cycle arrest. The activity and mRNA and protein expression of caspases 3, 8 and 9 were detected to determine the apoptotic pathway. Caspase expression was blocked by caspase inhibitors in order to observe whether the intrinsic or extrinsic pathway contributed to HepG2 cell apoptosis. The results revealed that the mRNA and protein expression of cyclins A and D1 was downregulated while the expression of P21^Waf/cip1^ was upregulated by VPA. The expression of cyclin E was only slightly affected by VPA. The mRNA and protein expression and activity of caspases 3 and 9 were upregulated by VPA. By contrast, inhibitors of caspases 3 and 9 could reverse cell apoptosis and there was no notable change in caspase 8 expression in any of these experiments. The intrinsic apoptosis pathway, but not the death receptor pathway, contributed to the induction of apoptosis in hepatocellular carcinoma cells. Furthermore, VPA could inhibit the proliferation of hepatocellular carcinoma cells by inducing G_1_ phase arrest and cell apoptosis. These effects were attributed to the change in the caspase level.

## Introduction

Histone acetylation is associated with the genesis and development of certain tumors and is regulated by histone acetyltransferase (HAT) and histone deacetylase (HDAC) (1,2). Thus, suppressing HDAC can be used as a novel antitumor therapy (3,4). HDAC inhibitors (HDACIs) are notable due to their antitumor function (5,6). However, numerous HDACIs that are currently used in the clinic, including trichostatin A (TSA), apicidin and suberoylanilide hydroxamic acid (SAHA), have been restricted due to toxicity and a short half-life (7). Valproate acid sodium (VPA), a short-chain fatty acid with the chemical name 2-sodium valproate, was demonstrated to be a specific HDAC inhibitor and has been used widely as an anticonvulsant drug with low toxicity and a long half-life (8).

Classical therapy for hepatocellular carcinoma, a malignant tumor that exhibits a quick progression, poor prognosis and high mortality rate, is unsatisfactory and novel treatment methods are required (9). In the present study, VPA was used to reverse the malignant phenotypes of hepatocellular carcinoma through regulating the level of histone acetylation, and the HDACI mechanism of VPA was determined. The apoptosis pathway of hepatocellular carcinoma HepG2 cells was also identified and, finally, the anticarcinoma effects of VPA on a hepatocellular carcinoma mouse model were estimated *in vivo*.

## Materials and methods

### Cell culture and induction

HepG2, BEL-7402 and SMMC-7721 cells (Cell Bank of Type Culture Collection of Chinese Academy of Sciences, Shanghai, China) were cultured in RPMI-1640 standard medium (Gibco Life Technologies) supplemented with 10% fetal bovine serum (Tianhang, Zhejiang, China), glutamine (Tianhang) and antibiotics (50 IU penicillin and 50 μg/ml streptomycin; Sigma-Aldrich, St. Louis, MO, USA) in a humidified 5% CO_2_ and air atmosphere at 37°C. Exponentially growing HepG2 cells were incubated in six-well plates at a concentration of 1×10^5^/ml. Subsequent to culturing at 37°C in 5% CO_2_ for 2 h, 3.0 mmol/l VPA (Sigma-Aldrich) was added. After a 48-h induction, the cells were harvested for the following experiments.

### Effect of VPA on HDAC activity and gene expression

#### HDAC activity

TheHepG2, BEL-7402 and SMMC-7721 cells (5×10^4^ /ml) were induced by 3.0 mmol/l VPA for 48 h. The cells were collected and 100 μg nuclear extract was used to detect the total HDAC activity using a colorimetric HDAC activity assay kit (BioVision, Inc., Milpitas, CA, USA), according to the manufacturer’s instructions.

#### mRNA expression of HDAC1

HDAC1 mRNA expression was detected by reverse transcription-polymerase chain reaction (RT-PCR). Total RNA was extracted from the cells using TRIzol reagent (Gibco Life Technologies, Carlsbad, CA, USA) and RT-PCR was performed. The PCR products were assayed by 1% agarose gel electrophoresis, visualized under a gel-image analysis system (Uvitec Ltd., Cambridge, Cambridgeshire, UK) and then analyzed using the UVIband image analyzer (Uvitec Ltd.). The relative intensity of objective HDAC1 mRNA was indicated by the ratio of the objective optical density (OD) to the OD for β-actin. The control cells were treated with the culture medium without VPA.

### Cell culture and proliferation assay

Exponentially growing HepG2, BEL-7402 and SMMC-7721 cells (0.1 ml) were incubated in 96-well plates at a concentration of 1×10^5^ cells/ml. Subsequent to culturing at 37°C in 5% CO_2_ for 2 h, 3.0 mmol/l VPA was added. After 48 h, the cell proliferation was assessed by 10 μl MTT (5 mg/ml). The control cells were cultured without VPA. The cell growth inhibition rate (%) was calculated as follows: (A_control cell_ − A_VPA-treated cell_) / A_control cell_ × 100, where ‘A’ is the absorbance.

### Cell cycle assay and expression of associated gene and protein

#### Cell cycle assay

In accordance with the assay results, the HepG2 cell line was used in the following detection. The HepG2 cells were induced using the aforementioned method with 3.0 mmol/l VPA for 48 h. The cells were then collected and washed twice in phosphate-buffered saline (PBS) and incubated overnight in cold 70% ethanol at 4 °C. Subsequently, the cells were rinsed with PBS and stained with propidium iodide (PI) working solution (0.2 mg/ml PI, 0.08 mg/ml ribonuclease A and 0.5 mg/ml trypsin inhibitor; Sigma) for 30 min at room temperature and in the dark. The stained nuclei were analyzed using flow cytometry (FCM; Beckman Coulter, Brea, CA, USA) and the cell cycle was analyzed by the MacCycle software (Beckman Coulter). The control cells were cultured without VPA.

#### mRNA expression of cyclins and P21^Waf/cip1^

HepG2 cells were induced and RT-PCR was performed using the aforementioned method to detect the expression of cyclin A, D1 and E and P21^Waf/cip1^.

#### Protein expression of cyclins and P21^Waf/cip1^

FCM was used to detect the protein expression of cyclins A, D1 and E and P21^Waf/cip1^. The primary antibodies used in this study were against cyclin A (mouse monoclonal anti-cyclin A antibody, 6E6; cat. no. MS-1062-S0,-S1; NeoMarkers, Fremont, CA, USA), cyclin D1 (mouse monoclonal anti-cyclin D1 antibody, DCS-6; cat. no. MS-210-P0,-P1; NeoMarkers), cyclin E (mouse monoclonal anti-cyclin E antibody, HE12; cat. no. MS-870-P0,-P1; NeoMarkers) and P21^Waf/cif1^ (mouse monoclonal anti-P21 antibody, F-5; cat. no. SC-6246; Santa Cruz Biotechnology, Inc., Santa Cruz, CA, USA). The monoclonal antibodies were diluted (1:40) using PBS solution with 0.1% sodium azide. The secondary antibody was FITC-rabbit polyclonal anti-mouse IgG (1:40; H+L; Signalway Antibody, College Park, MD, USA). Briefly, 5×10^6^ HepG2 cells were collected and washed following exposure to 3.0 mmol/l VPA for 48 h. The cells were mixed with 1,000 μl permeabilization buffer and incubated for 15 min at room temperature. The supernatant liquor was replaced by 100 μl permeabilization buffer following centrifugation at 2,000 × g for 10 min, and the cells were suspended and mixed with 5 μl (1 μg) monoclonal antibodies for cyclins A, D1 and E and P21^Waf/cip1^. After 30 min, the cells were washed twice with PBS. The cells were then mixed with 100 μl (2.5 μg) secondary antibody at room temperature and in the dark for 30 min. Subsequent to the cells being washed twice and resuspended in PBS, protein expression was analyzed by FCM and the mean fluorescence intensity exponent (MFI) was calculated.

### Effect on HepG2 cell apoptosis and caspases by VPA

#### Apoptosis assay

VPA (3.0 mmol/l) was used to induce HepG2 cell apoptosis. Briefly, following 48 h of induction, the cells were collected, washed once with PBS, stained with Annexin V/PI according to the manufacturer’s instructions, and then analyzed using FCM (Beckman Coulter) and MacCycle software. The control cells were treated with the culture medium without VPA.

#### Caspase activity

To determine the pathway through which HepG2 cell apoptosis is induced by VPA, the activity of caspases 3, 8 and 9 was measured using the caspase activity detection kit (Nanjing KeyGen Biotech Co., Ltd., Nanjing, China). Briefly, HepG2 cells were induced and collected, and were washed once using PBS. Protein was extracted from the cells using cell lysates contained within the kit, and the activity of caspases 3, 8 and 9 was measured according to the manufacturer’s instructions. A_405_ was used to denote caspase activity.

#### Caspase blocking assay

Caspase inhibitors were used to block caspase expression. Briefly, HepG2 cells were induced by 3.0 mmol/l VPA combined with 40 μmol/l caspase 3 inhibitor Z-DEVD-FMK, caspase 8 inhibitor Z-IETD-FMK and caspase 9 inhibitor Z-LEHD-FMK, respectively (BioVision, Inc.). Apoptosis was detected using the aforementioned method following 48 h. Cells induced by VPA alone were used as the positive control, and normal control cells were cultured with culture medium.

#### Protein expression of caspases

The protein expression of caspases 3, 8 and 9 was detected using the aforementioned FCM technique.

### Statistical analysis

Each experiment was performed at least in triplicate. All the results were expressed as the mean ± standard deviation. The data were analyzed by the Student’s unpaired t-test. P<0.05 and P<0.01 were considered to indicate a statistically significant difference.

## Results

### Effect of VPA on HDAC activity and gene expression

**Figure 1:**
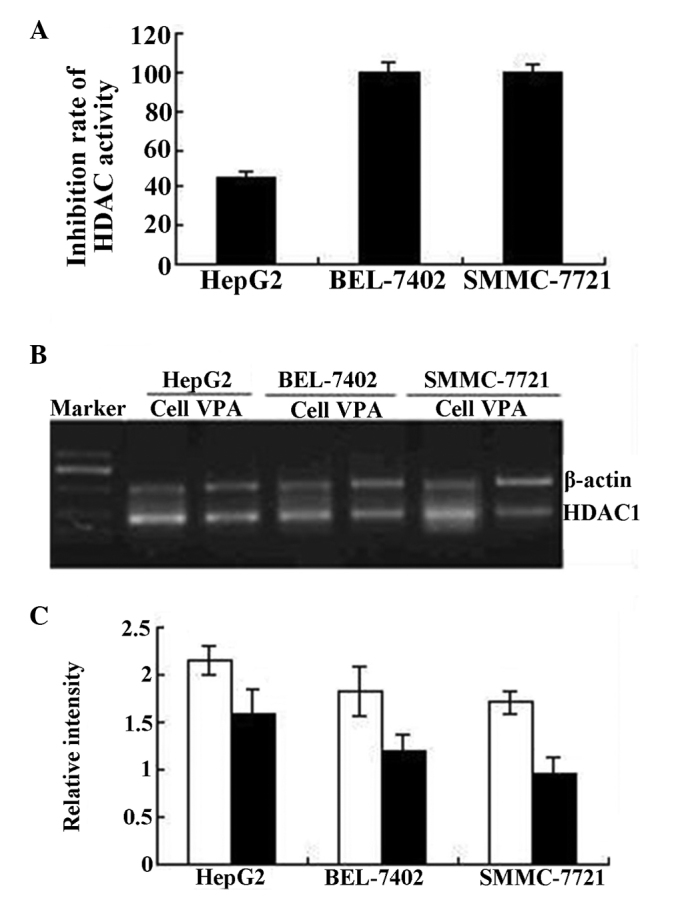
Effect of VPA on HDAC activity and gene expression. HepG2, BEL-7402 and SMMC-7721 cells (5×10^4^ /ml) were induced by 3.0 mmol/l VPA for 48 h. Nuclear extract (100 μg) was used to detect the total HDAC activity by the colorimetric HDAC activity assay kit. mRNA expression of HDAC1 was detected by RT-PCR. The relative density of objective HDAC1 mRNA was indicated by the ratio of OD_objective_ to OD_β-actin_. (A) Effect of VPA on HDAC activity, (B) profile of HDAC1 mRNA expression and (C) relative intensity of HDAC1 mRNA. White bars, control cells; black bars, VPA-induced cells. VPA, valproate acid sodium; HDAC, histone deacetylase; RT-PCR, reverse transcription-polymerase chain reaction; OD, optical density.

The total HDAC activity of HepG2, BEL-7402 and SMMC-7721 cells was markedly inhibited following 48 h of treatment with VPA. HDAC activity in the BEL-7402 and SMMC-7721 cells was completely inhibited (100 and 99.5%, respectively) by 3.0 mmol/l VPA, while 45.1% of HDAC activity in HepG2 cells was inhibited. The RT-PCR results revealed that the expression of HDAC1 mRNA in HepG2, BEL-7402 and SMMC-7721 cells was also inhibited by VPA, as described in Fig. 1.

### Effect of VPA on the proliferation of HepG2, BEL-7402 and SMMC-7721 cells

**Figure 2:**
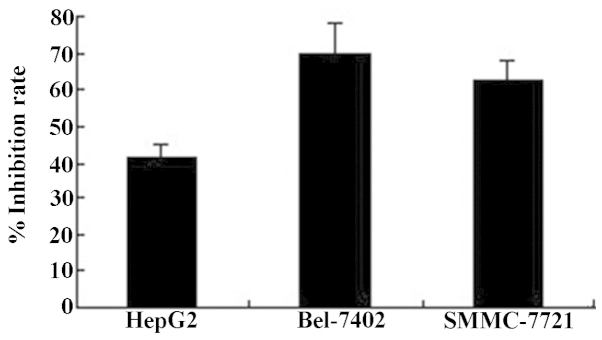
Effect of VPA on viability and proliferation of hepatocellular carcinoma HepG2, BEL-7402 and SMMC-7721 cells. The cells (5×10^4^ cells/ml) were incubated with 3.0 mmol/l VPA for 48 h. The proliferation of cells was determined by MTT assay. The data were obtained from the mean of three independent experiments, in which the determinations were performed in triplicate and the cell proliferation inhibition rate was calculated. VPA, valproate acid sodium.

Compared with the control cells, the proliferation of HepG2, BEL-7402 and SMMC-7721 cells was evidently inhibited by 3.0 mmol/l VPA, with an inhibition rate of 41.6, 62.6 and 69.8%, respectively (Fig. 2).

### Effect of VPA on the cell cycle of HepG2 cells

#### Cell cycle profile

**Figure 3:**
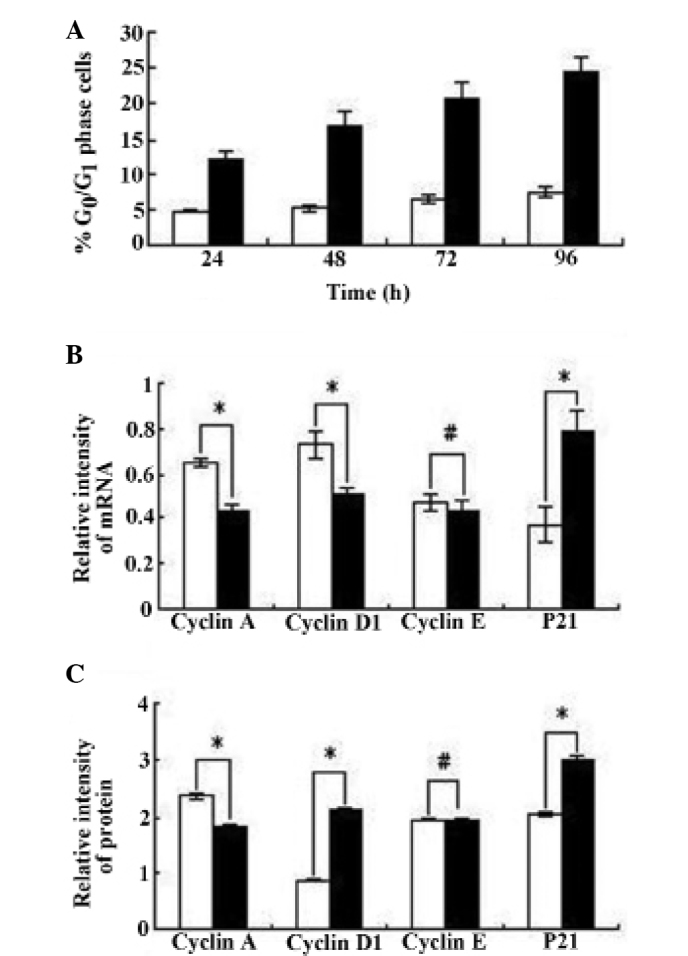
Effect of VPA on the cell cycle of HepG2 cells. The HepG2 cells (1×10^5^ cells/ml) were incubated with 3.0 mmol/l VPA. The cell-cycle profile were measured by propidium iodide staining and FACS analysis. The data are shown as the percentage distribution of cells in the G_0_/G_1_ phase. To analyze the effect of VPA on mRNA and the protein expression of cyclins A, D1 and E and P21^Waf/cip1^, the HepG2 cells were induced by 3.0 mmol/l VPA for 48 h. mRNA expression was detected by reverse transcription-polymerase chain reaction, and protein expression was measured by flow cytometry analysis. (A) Cell-cycle profiles for 24, 48, 72 and 96 h. P<0.01, as compared with each experimental group. (B) Relative intensity of mRNA expression. (C) Relative intensity of protein expression. ^*^P<0.01 and ^#^P>0.05. White bars, control cells; black bars, VPA-induced cells. VPA, valproate acid sodium.

According to the inhibition of cell proliferation and the cell cycle profile of HepG2 cells revealed by FCM assay, there was no evident change in any phase of the cell cycle in the control cells, but there was an increased proportion of VPA-treated cells in the G_0_/G_1_ phase (P<0.01; Fig. 3A).

#### mRNA expression of cyclins A, D1 and E, and P21^Waf/cip1^

mRNA expression was detected by RT-PCR assay. Fig. 3B shows that compared with the control cells, the mRNA expression of cyclins A and D1 was downregulated and the expression of P21^Waf/cip1^ was markedly upregulated in VPA-treated cells (P<0.01). However, the expression of cyclin E was not significantly affected by VPA (P>0.05).

#### Protein expression of cyclins A, D1 and E and P21^Waf/cip1^

Fig. 3C shows that the protein expression detected by the FCM assay was in accordance with the mRNA expression. The protein expression of cyclins A and D1 was downregulated and P21^Waf/cip1^ expression was markedly upregulated in cells incubated with VPA (P<0.01). However, cyclin E expression demonstrated no significant difference between the VPA-treated and control cells (P>0.05).

### Effect on apoptosis of HepG2 cells by VPA

#### HepG2 cell apoptosis induced by VPA

**Figure 4:**
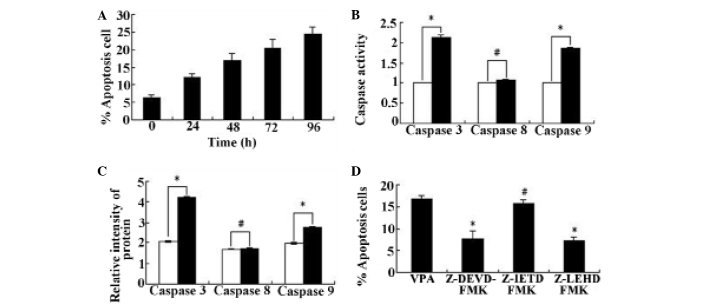
HepG2 apoptosis assay and apoptosis pathway detection. The HepG2 cells were induced by 3.0 mmol/l VPA, then cell apoptosis was detected by flow cytometry using an Annexin V/propidium iodide kit. The activity of caspases 3, 8 and 9 was detected using caspase activity detecting kits, and the activity of the control cell was denoted as 1. Caspase protein expression was detected by flow cytometry analysis and the mean fluorescence intensity exponent was calculated. To detect the effect of caspase inhibitors on the apoptosis-inducing function of VPA, the HepG2 cells were induced by a combination of 3.0 mmol/l VPA and 40 μmol/l of the caspase 3, 8 and 9 inhibitors Z-DEVD-FMK, Z-IETD-FMK and Z-LEHD-FMK, respectively, for 48 h, and the results were indicated by the percent of apoptotic cells. The experiment was repeated two times. (A) Apoptosis profile of HepG2 cells treated with VPA for 24, 48, 72 and 96 h. (B) Caspase activity inhibited by VPA. ^*^P<0.01 and ^#^P>0.05. (C) Caspase protein expression affected by VPA. ^*^P<0.01 and ^#^P >0.05. (D) Effect of caspase inhibitors on HepG2 cell apoptosis. ^*^P<0.05 and ^#^P>0.05. White bars, control cells; black bars, VPA-induced cells. VPA, valproate acid sodium.

Fig. 4A shows that, as a HDAC inhibitor, the mechanism of proliferation inhibition exerted by VPA on HepG2 cells was detected. Compared with the control cells, the HepG2 cells treated with 3.0 mmol/l VPA for 48 h demonstrated evident apoptosis and the apoptosis rate increased from 6.25 to 29.5% (P<0.01).

#### Effect of VPA on caspase activity

Fig. 4B shows that the activity of caspases 3 and 9 was increased by 113.0% and 86%, respectively, by VPA, which is significantly different compared with the control group (P<0.01). However, caspase 8 activity exhibited no clear difference between VPA-treated and control cells (P>0.05).

#### Protein expression of caspases

The protein expression of caspases in HepG2 cells was detected by FCM analysis and was indicated by MFI. In agreement with the caspase activity assay, the MFI of caspases 3 or 9 in HepG2 cells treated with VPA was higher compared with untreated cells (P<0.01), which means the protein expression of caspases 3 and 9 in HepG2 cells was markedly upregulated by VPA. However, caspase 8 expression was only slightly affected by VPA (P>0.05; Fig. 4C).

#### Caspase-blocking assay

Similar to the activity and protein expression of the caspases, Fig. 4D shows that the VPA-induced apoptosis of HepG2 cells could be reduced by caspase 3 and 9 inhibitors through blocking the expression of caspase proteins. The cell apoptosis rate was 16.9% when the cells were induced by VPA alone. However, the apoptosis rate was only 7.6% when induced by VPA and the caspase 3 inhibitor Z-DEVD-FMK, and was 7.2% when induced by VPA and the caspase 9 inhibitor Z-LEHD-FMK (P<0.05). By contrast, there was no evident change in the cell apoptosis rate when induced by VPA and the caspase 8 inhibitor Z-IETD-FMK, with a rate of 15.8% (P>0.05).

## Discussion

In the 1950s, Cruft reported that histone could bind to DNA and change the transcription activity (10). From then on, histones became a hot research point (11). High expression of HDACs is closely correlated with tumorigenesis and tumor development (12). VPA has been previously demonstrated to be a specific HDACI, but its antineoplastic function has not been widely noticed (13).

In the present study, VPA was demonstrated to be an HDACI, as HDAC activity and the HDAC1 gene expression of hepatocellular carcinoma cells was inhibited by it. As a result, cell proliferation inhibition by VPA was detected.

From the inhibition results of cell proliferation and HDAC activity, it was observed that BEL-7402 and SMMC-7721 cells were more sensitive than HepG2 cells to VPA. Therefore, HepG2 cells were used in the subsequent experiments. If the malignant phenotype of HepG2 cell can be reversed by VPA, the malignant phenotype of SMMC-7721 and BEL-7402 cells should also be reversed.

Abnormal expression of cyclin proteins is associated with the genesis and prognosis of certain tumors (14,15). The expression of cyclins D1, A and E significantly increases in tumor tissue compared with normal tissue (1,16). Genetic transcription and protein expression of cyclin A, D and E can be blocked by P21. The P21^Waf/cip1^ gene is a cyclin-dependent kinase (CDK) inhibitor that is important for repairing DNA injury and correcting DNA replication (17). If the DNA is damaged at the G_1_ phase, P21^Waf/cip1^ genetic transcription is activated and binds to the cyclin and CDK, resulting in the cyclin-CDK compound losing its kinase activity. Therefore, cell-cycle arrest at the G_1_ phase and DNA replication is inhibited, so cell growth is interrupted. It has been reported that histone deacetylation is the key mechanism of P21^Waf/cip1^ inactivation in gastric carcinoma cell lines (18–21). The present results revealed that, following treatment with VPA for 48 h, the cell cycle was arrested at the G_0_/G_1_ phase and the mRNA and protein expression of cyclin A and D1 was downregulated in HepG2 cells, while the expression of P21^Waf/cip1^ was upregulated. However, cyclin E was only marginally affected by VPA. The VPA-induced G_1_-phase arrest in HepG2 cells may occur through the following pathways, individually or synergistically. VPA may upregulate P21^Waf/cip1^ mRNA and protein expression, which binds to CDKs competitively with cyclins and inhibits various cyclin-CDK compounds. VPA may also downregulate cyclin D1 mRNA and protein expression, leading to decreased activity of the cyclinD-CDK4/CDK6 pathway, so cell proliferation does not skip the G_1_ phase, or VPA may downregulate cyclin A mRNA and protein expression, followed by a reduction in the synthesis of cyclin A-CDK2 and cyclin A-CDK1 compounds. Therefore, DNA synthesis of S phase cells may reduce, and the cells are prevented from switching to M phase from G_2_ phase and, as a result, the cells arrested at the G_1_ phase. In the majority of cases, cell cycle arrest is the directional step prior to cell apoptosis.

In view of apoptosis as one mechanism for antitumor proliferation, the present study focused on the function that VPA induces apoptosis in hepatocellular carcinoma cells, and aimed to determine the contribution of the intrinsic or extrinsic pathways to the apoptosis. Firstly, the VPA-induced apoptosis of HepG2 cells was identified by FCM using an Annexin V/PI apoptosis detection kit. Caspases are the center components of apoptosis, and the cascade reaction of caspases is the activator (22,23). Caspase 3 is the most important effector molecule in the caspase family and is located at the termini of the apoptosis process, termed the apoptosis executor. Caspases 8 and 9 are promoters of the intrinsic and extrinsic apoptosis pathway, respectively. The present results revealed that, following treatment with VPA, the activity and protein expression of caspases 9 and 3 was markedly upregulated, but caspase 8 expression was not evidently changed. Furthermore, HepG2 cell apoptosis could be reduced by inhibitors of caspases 3 and 9, but not by caspase 8 inhibitors. All these indicate that VPA could induce the apoptosis of HepG2 cells by activating the intrinsic or mitochondrion pathway.

The acetylation level of the histone N terminal can alter the condition of chromatin by interfering with the affinity of histones for DNA, or by disturbing the combination of transcription factors and DNA sequence (24). The regulatory role of acetylation in gene expression is similar to the DNA genetic code, and so the role of HDACIs was observed in tumor therapy. The present study revealed that regulating histone acetylation with VPA can markedly reverse the malignant phenotype of hepatoma carcinoma cells, confirming that it can be widely applied in hepatoma treatment. However, it was the intrinsic apoptosis pathway that contributed to the induction of apoptosis by VPA in hepatocellular carcinoma cells.
